# Epilepsy: Its Pathology and Treatment

**Published:** 1891-06

**Authors:** 


*Epilepsy: its Pathology and Treatment.* By HOBART AMORY HARE, M.D. Philadelphia: F. A. DAVIS. 1890.

The essay on Epilepsy, to which was awarded the prize by the Académie Royale de Médecine de Belgique, Dr. Hare has published in the Physicians' and Students' Ready Reference series. Like most prize essays, the work is mainly based upon the observations of others, and in some sections seems to lack that spontaneity and coherence found in the original work of an independent investigator. Not that we wish to suggest that Dr. Hare has not himself done good work, the results of which are incorporated in the text of the book; but, in order to obtain statistics dealing with cases in large numbers, it is necessary to mass together the results obtained by many observers. Dr. Hare has carefully consulted all that has been recently published on Epilepsy, and gives abundant references in foot-notes to the cosmopolitan literature of the subject. The chapter on Pathology is mainly devoted to expressing his own views on this topic, and reads therefore with a greater homogeneity than some other parts of the book. Taken as a whole the work is a good and succinct compendium of our present knowledge of Epilepsy, for which we thank Dr. Hare, and at the same time offer him our congratulations upon his being elected to succeed so distinguished a predecessor.

